# Navigating the distal vasculature: Challenges and lessons learned from failed Thrombectomy trials

**DOI:** 10.1177/15910199251365526

**Published:** 2025-08-14

**Authors:** Peter B. Sporns, Mohammad Almohammad, Jens Minnerup, Thi Dan Linh Nguyen-Kim, Jens Fiehler, Lars Timmermann, André Kemmling

**Affiliations:** 1 Department of Radiology and Neuroradiology, 30262 Stadtspital Zürich, Zürich, Switzerland; 2 Department of Neuroradiology, University Hospital Basel, Basel, Switzerland; 3 Department of Diagnostic and Interventional Neuroradiology, 37734 University Medical Center Hamburg-Eppendorf, Hamburg, Germany; 4 Department of Neuroradiology, 61061 University Hospital Marburg, Marburg, Germany; 5 Department of Neurology, University of Lübeck and 37734 University Hospital Schleswig-Holstein, Lübeck, Germany; 6 Department of Neurology, 61061 University Hospital Marburg, Marburg, Germany

**Keywords:** EVT, DISTAL, thrombectomy, MeVO, DMVO

## Abstract

**Background:**

While endovascular thrombectomy (EVT) is firmly established for large vessel occlusion stroke, recent enthusiasm for extending EVT to distal medium vessel occlusions (DMVOs) has been tempered by the neutral results of three major randomized controlled trials: DISTAL, ESCAPE-MeVO, and DISCOUNT.

**Objective:**

To critically examine why EVT failed to demonstrate benefit in DMVO trials, assess the associated procedural and clinical challenges, and explore potential future directions for safe and effective treatment in distal cerebrovascular territories.

**Methods:**

This review synthesizes the key findings of recent DMVO thrombectomy trials and contextualizes them within the anatomical, technical, and clinical limitations specific to distal interventions. It further highlights innovations of devices and distal EVT techniques as possible solutions.

**Findings:**

The trials showed no functional benefit of EVT over best medical therapy for unselected DMVO patients and raised safety concerns, including partially increased rates of symptomatic intracranial hemorrhage and mortality in the EVT arms. Contributing factors include the fragility and tortuosity of distal vessels, suboptimal device compatibility, variability in operator experience, and potential limitations in imaging and patient selection. Preliminary data suggest that refined techniques may mitigate risks in very distal occlusions.

**Conclusions:**

Routine EVT for DMVO stroke cannot be recommended based on current evidence. Future research must prioritize patient stratification, dedicated distal devices, and procedural innovation to safely extend thrombectomy into distal territories.

## Introduction

Endovascular thrombectomy (EVT) has revolutionized the treatment of acute ischemic stroke caused by large vessel occlusion (LVO) in the anterior circulation. Landmark randomized controlled trials (RCTs) published since 2015, such as MR CLEAN, ESCAPE, EXTEND-IA, SWIFT PRIME, and REVASCAT, conclusively demonstrated the overwhelming benefit of rapid mechanical clot removal in addition to best medical management, significantly improving functional outcomes for patients with LVO strokes.^1–3^ Subsequent studies further expanded the indications, showing benefit in extended time windows and for patients with larger ischemic cores, solidifying EVT as the standard of care for eligible LVO patients.^4,5^

The profound success observed in LVO naturally spurred interest in extending the benefits of EVT to patients suffering strokes from more distal occlusions, specifically involving medium vessel occlusions (MeVO) or distal medium vessel occlusions (DMVOs), such as those in the M2 or M3 segments of the middle cerebral artery, or equivalent vessels in the anterior and posterior cerebral arteries.^6^ These DMVOs account for a substantial proportion of acute ischemic strokes, estimated between 25% and 40%, and can lead to significant disability despite often presenting with lower initial National Institutes of Health Stroke Scale (NIHSS) scores compared to LVOs.^7^ The rationale was compelling: if removing larger clots was beneficial, perhaps removing smaller, more distal clots could prevent devastating infarcts in eloquent territories and improve outcomes for this sizable patient group.

However, this enthusiasm has been significantly tempered by the recent results of three major randomized RCTs specifically designed to test the efficacy of EVT for DMVOs. Presented in early 2025, the DISTAL, ESCAPE-MeVO, and DISCOUNT trials consistently failed to demonstrate a clinical benefit for EVT added to best medical therapy (BMT) compared to BMT alone.^8–10^ In fact, the DISTAL and ESCAPE-MeVO trials showed numerically higher rates of adverse events, including mortality and symptomatic intracranial hemorrhage (sICH), in the thrombectomy arms, while the interim analysis of DISCOUNT suggested potentially worse outcomes with EVT.^8,10,11^ These unexpected and disappointing findings represent a major setback and necessitate a critical re-evaluation of the role of thrombectomy in this patient population.

This review discusses potential reasons underpinning the failure of these recent distal thrombectomy trials. Among these reasons are inherent procedural challenges and increased risks associated with intervening in smaller more fragile distal vessels, the complexities of patient selection, the impact of advanced imaging techniques (or lack thereof), and the potentially high efficacy of modern BMT in the control groups. Possible solutions may be found in specialized techniques developed for very distal occlusions, such as the Soft Partial Release of Non-aggressive Stent Retriever (SPORNS) technique.^12^ Niche approaches might hold promise where broader application has failed, and point to future directions for research in this challenging field.

## Summary of key distal medium vessel occlusion trials

The recent neutral findings from major RCTs investigating EVT for distal DMVOs stand in stark contrast to the established success in LVOs. Three key trials—DISTAL, ESCAPE-MeVO, and DISCOUNT—reported concurrently in early 2025, provided high-quality evidence that has significantly challenged the presumed benefits of extending EVT into these smaller vessels.^8–10^

The DISTAL trial, presented by Fischer and Psychogios, randomized 543 patients (median age 77 years, median baseline NIHSS score of 6) with acute ischemic stroke due to an isolated occlusion in the M2 or M3 segments of the middle cerebral artery, or distal segments of the anterior or posterior cerebral arteries, presenting within 24 h of onset.^10^ Patients were assigned to receive either EVT plus BMT (BMT), including intravenous thrombolysis where appropriate (received by ∼65%), or BMT alone. The primary outcome, the distribution of modified Rankin Scale (mRS) scores at 90 days, showed no significant difference between the two groups (common odds ratio for improvement 0.90; 95% CI 0.67–1.22). Importantly, safety outcomes raised concerns, with numerically higher rates of all-cause mortality at 90 days (15.5% in the EVT group vs 14.0% in the BMT group) and sICH within 24 h (5.9% vs 2.6%) observed in the thrombectomy arm.^10^

Similarly, the ESCAPE-MeVO trial, led by Hill and Goyal, enrolled 530 patients (median age 75 years) across multiple countries who presented within 12 h of stroke onset with medium vessel occlusions (defined slightly differently but encompassing similar distal territories) and favorable baseline imaging.^8^ Patients were randomized to EVT plus usual care or usual care alone. The primary outcome, the proportion of patients achieving an excellent functional outcome (mRS score 0–1) at 90 days, was nearly identical between the groups (41.6% in the EVT arm vs 43.1% in the control arm; adjusted rate ratio 0.95; 95% CI 0.79–1.15). Again, safety signals were concerning, with higher rates of 90-day mortality (13.3% vs 8.4%) and sICH (5.4% vs 2.2%) associated with the endovascular procedure.^8^

The DISCOUNT trial, presented by Clarençon, focused on distal vessel occlusions in the anterior or posterior circulation confirmed on imaging, with patients randomized within 8 h of onset (or within 24 h if no hyperintense signal on FLAIR).^11,13^ An interim analysis of the first 163 patients (of a planned 488) revealed a concerning trend towards worse outcomes in the thrombectomy group. The proportion of patients achieving an mRS score of 2 or less at 90 days was substantially lower in those undergoing thrombectomy compared to those receiving BMT alone (60% vs 77%).^11^ Although an interim analysis, this finding added significant weight to the negative results from DISTAL and ESCAPE-MeVO.

Taken together, these three methodologically rigorous trials consistently failed to demonstrate any functional benefit for EVT when applied broadly to patients with DMVO strokes compared to modern BMT. Furthermore, the recurring safety signals, particularly the increased risk of sICH and potentially mortality, strongly suggest that the procedural risks associated with intervening in these smaller, more distal vessels may outweigh any potential benefits for the overall population studied.^8–10^ This collective evidence necessitates a pause and critical examination of why these trials failed.

## Procedural challenges and risks in distal vasculature

One primary hypothesis for the failure of EVT in DMVOs centers on the inherent difficulties and heightened risks associated with navigating and intervening within the distal cerebral vasculature. Unlike the relatively larger and more accessible proximal arteries involved in LVO strokes, the target vessels in DMVOs (such as M2, M3, A2, A3, P2, P3 segments) present distinct anatomical challenges.^6,7^ These vessels are characterized by significantly smaller diameters, increased tortuosity, more complex branching patterns, and inherently greater fragility compared to their proximal counterparts.^14^

These anatomical features translate directly into increased procedural risks during thrombectomy attempts. Navigating microcatheters and thrombectomy devices through these narrow and winding pathways is technically demanding and carries a higher likelihood of iatrogenic complications. Vessel perforation, dissection, or vasospasm can occur more readily, potentially leading to devastating hemorrhagic complications or worsening ischemic injury.^14,15^ The consistently higher rates of sICH observed in the EVT arms of the DISTAL, ESCAPE-MeVO, and DISCOUNT trials (e.g. 5.9% vs 2.6% in DISTAL, 5.4% vs 2.2% in ESCAPE-MeVO) lend strong support to the notion that the procedure itself introduces significant risks in this population.^8,10,11^ Even reaching the occlusion site can be challenging, potentially prolonging procedure times and increasing the risk of complications without successful recanalization.

Furthermore, the thrombectomy devices currently available, including stent retrievers and aspiration catheters, were largely developed and optimized for LVOs. Their size, stiffness, and mechanism of action may be less suitable or even hazardous when applied to smaller, more delicate distal vessels.^15^ The force required to deploy a stent retriever or the suction applied during aspiration might be sufficient to damage the vessel wall or dislodge atheroma, leading to distal embolization or hemorrhage. While smaller, more trackable devices are emerging, the trials largely utilized existing LVO technology, which may have contributed to the unfavorable risk-benefit ratio observed. Finally, operator experience likely plays a significant role. Performing thrombectomy in distal vessels is technically more challenging and may have a steeper learning curve, potentially influencing outcomes, especially outside highly specialized centers.

## Patient selection, imaging, and control group factors

Beyond procedural difficulties, the neutral results of the DMVO thrombectomy trials may also stem from complexities in patient selection, limitations in imaging assessment, and the effectiveness of the comparator treatment, BMT (Table 1). DMVO strokes represent a heterogeneous group, encompassing a wide range of clinical severities, infarct volumes, and underlying causes.^7^ The inclusion criteria used in DISTAL, ESCAPE-MeVO, and DISCOUNT, while aiming for consistency, inevitably enrolled a broad spectrum of patients. It is plausible that within this mixed population, only a specific subgroup stood to benefit significantly from EVT, while others either derived no benefit or were potentially harmed, thus diluting or negating any overall positive effect.^9,16^ For instance, patients with smaller initial stroke severity (as reflected by lower median NIHSS scores in DISTAL compared to typical LVO trials) or those with robust collateral circulation might have had good outcomes with BMT alone, leaving little room for EVT to demonstrate superiority.^10^

**Table 1.** Comparative summary of DISTAL, ESCAPE-MeVO, and DISCOUNT trials.

Category	DISTAL	ESCAPE-MeVO	DISCOUNT
**Trial design**	Assessor-blinded, randomized, pragmatic, multicenter	PROBE design: multicenter, randomized, open-label, blinded outcome eval	Randomized controlled trial (RCT), presented at ISC
**Sample size**	543 (271 EVT, 272 control)	530 (255 EVT, 275 control)	161 (64 EVT, 97 control)
**Primary outcome**	mRS shift analysis at 90 days	mRS 0–1 at 90 days	mRS 0–2 at 90 days
**NIHSS inclusion**	≥4 or disabling symptoms	>5 or 3–5 with disabling symptoms	≥5 or significant aphasia
**Time window**	≤24 h (with imaging mismatch after 6 h)	≤12 h	≤8 h
**Occlusion locations**	Nondominant/codominant M2/M3/M4 MCA, A1–A3 ACA, P1–P3 PCA	M2/M3 MCA, A2/A3 ACA, P2/P3 PCA	Distal M2/M3 MCA, A1/A2/A3 ACA, P1/P2/P3 PCA
**Devices allowed**	Any approved EVT device	Solitaire X required as first-line device	Trevo, CatchView Mini, Q3–5, etc.
**Intravenous thrombolysis (%)**	62.0% (EVT), 68.8% (control)	56.5% (EVT), 60.2% (control)	70% (EVT), 71% (control)
**Median NIHSS**	6 (IQR 5–9)	EVT: 8 (IQR 6–11), Control: 7 (IQR 5–11)	8 (IQR 6–11)
**Median age (years)**	77 (IQR 68–83)	EVT: 74 (IQR 63–82), Control: 76 (IQR 65–83)	75 (EVT), 72 (control)
**Successful reperfusion (%)**	71.7%	75.1%	77%
**Symptomatic ICH (%)**	EVT: 5.9%, Control: 2.6%	EVT: 5.4%, Control: 2.2%	EVT: 12%, Control: 6%
**Mortality at 90 days (%)**	EVT: 15.5%, Control: 14.0%	EVT: 13.3%, Control: 8.4%	EVT: 3%, Control: 7%
**Excellent outcome (mRS 0–1)**	EVT: 34.7%, Control: 37.5%	EVT: 41.6%, Control: 43.1%	Not reported
**Functional independence (0–2)**	EVT: 56.5%, Control: 54.7%	EVT: 54.1%, Control: 58.8%	EVT: 60%, Control: 77%
**Key exclusions**	Dominant M2 occlusion, large infarct, poor prognosis	ASPECTS ≤8, nursing care dependency, major comorbidities	Large core infarct, spontaneous recanalization, mRS >1

EVT: endovascular thrombectomy; MeVO: medium vessel occlusion; mRS: modified Rankin Scale.

Moreover, it should be considered that patients presenting with more severe symptoms may have been preferentially treated outside of the trials, in light of the overwhelming existing evidence supporting EVT for LVO strokes. This selection bias could have influenced trial outcomes by excluding those most likely to benefit from EVT, thereby skewing the trial populations toward those with milder presentations and diminishing the observable treatment effect.

Imaging plays a crucial role in stroke treatment decisions, particularly in selecting patients for reperfusion therapies. However, accurately assessing the ischemic core and penumbra in distal territories using standard imaging techniques (CT or MRI perfusion) can be more challenging than in LVO.^6^ Identifying the precise location of the occlusion and determining the volume of salvageable tissue might be less reliable, potentially leading to the inclusion of patients with already established large distal infarcts or those with minimal salvageable tissue, neither of whom would be expected to benefit significantly from reperfusion. Furthermore, the assessment and impact of collateral circulation, a key determinant of outcome and treatment response, might differ in DMVO compared to LVO, and its role in patient selection for distal EVT requires further clarification.^16^

Finally, the effectiveness of the control arm—BMT—cannot be overlooked. Modern BMT, particularly when including timely intravenous thrombolysis (administered to a significant proportion of patients in the trials, e.g. ∼65% in DISTAL and ∼58% across arms in ESCAPE-MeVO), can be highly effective in achieving recanalization and promoting good outcomes, especially for smaller or more distal clots.^8,10^ The relatively high rates of good functional outcomes observed in the BMT arms of these trials (e.g. 43.1% mRS 0-1 in ESCAPE-MeVO control, 77% mRS 0-2 in DISCOUNT control interim analysis) suggest that BMT sets a high bar for EVT to surpass.^8,11^ It is possible that for many patients included in these trials, the incremental benefit offered by EVT over already effective medical management was minimal and insufficient to overcome the inherent procedural risks.

## Future perspectives

**Figure 1.**
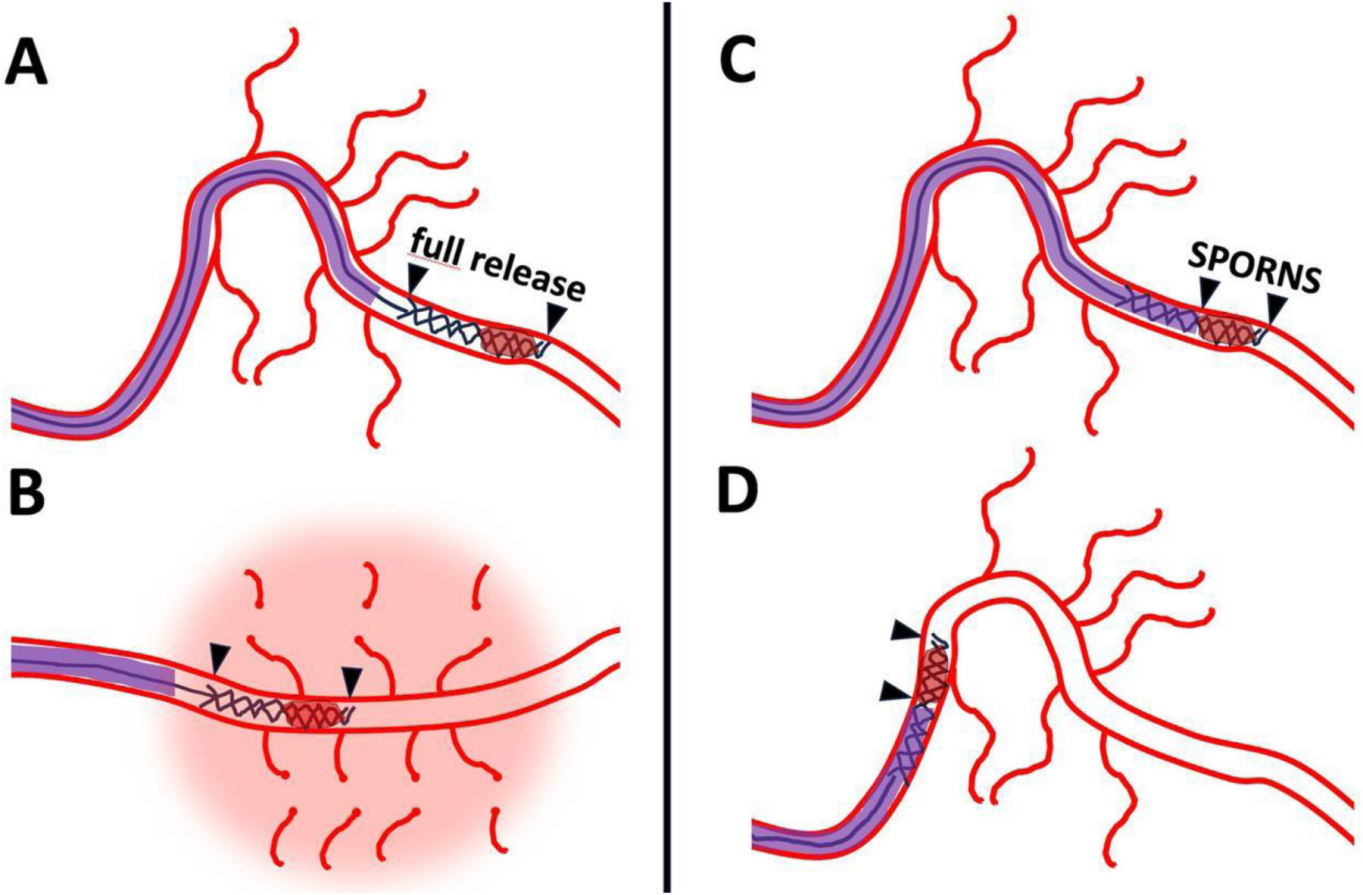
Challenges of distal thrombectomy. Illustration of a potential challenge of distal EVT with stent retrievers. (A) It shows full deployment of stent retriever in distal arterial branch, here M3 segment of the middle cerebral artery. (B) It illustrates straightening of the curve in the Sylvian fissure due to high friction of the stent retriever which is in full contact with the arterial wall. Small perforators are sheared off leading to subarachnoid hemorrhage. In contrast, (C) shows soft partial release of the stent retriever using the SPORNS technique, leading to minimized friction avoiding hemorrhagic complications (D). EVT: endovascular thrombectomy; SPORNS:.

Amidst the largely negative results from broad DMVO trials, specific techniques tailored for the unique challenges of the distal vasculature warrant consideration. One such approach is the SPORNS technique,^12^ that was specifically developed for thrombectomy in very distal arterial occlusions, targeting segments like M3/M4, A2/A3, and P1-P3, which may represent a subset distinct from the broader MeVO populations included in DISTAL and ESCAPE-MeVO. The core principle involves a modified deployment of a non-aggressive stent retriever, utilizing a soft partial release mechanism intended to minimize friction and mechanical stress on the delicate vessel walls and perforating arteries, thereby aiming to reduce the risk of hemorrhagic complications^12^ (Figure 1).

Preliminary data presented from a retrospective study involving 54 patients treated with the SPORNS technique across two centers showed promising results within its specific niche. High rates of successful recanalization (eTICI 2c/3 in 92% of patients) were reported, accompanied by substantial clinical improvement (median NIHSS decrease from 9 to 3) and a high proportion of good functional outcomes (mRS 0-2 in 75% at 90 days). Crucially, the reported rate of sICH was low (1 patient, 4%), with only a small number (8%) experiencing minor subarachnoid hemorrhage.^12^ While this is retrospective data from select centers and requires prospective validation, it suggests that specialized techniques focusing on atraumatic clot removal might hold potential for improving the safety profile of thrombectomy in the most distal, challenging locations.

In addition to stent retriever-based approaches, the use of small aspiration catheters designed for primary aspiration of DMVOs is gaining attention. Recent studies suggest that newer low-profile aspiration devices, when used alone or in combination with stent retrievers, may provide effective clot retrieval in distal arteries while minimizing vessel trauma.^17^ The flexibility and navigability of these catheters make them particularly suitable for tortuous distal segments, supporting their potential role in tailored DMVO interventions.

Another emerging method is the Quattro technique, which involves a four-component combined strategy optimized for DMVOs.^18^ This includes the simultaneous use of aspiration, stent retriever engagement, and distal access catheter support to maximize clot engagement and retrieval. Preliminary observations suggest it may improve efficacy and safety in complex distal occlusions, though robust prospective data are still needed.

However, it is crucial to contextualize the SPORNS findings. This technique targets a potentially more selective group of very distal occlusions than the broader MeVO populations studied in the large RCTs. The promising results do not invalidate the negative findings of DISTAL, ESCAPE-MeVO, and DISCOUNT for general DMVO thrombectomy but rather highlight that a “one-size-fits-all” approach is likely inappropriate.^8,10,11^ Success in distal thrombectomy may require not only specialized techniques but also meticulous patient selection.

Recently, subanalyses of DISTAL and ESCAPE-MeVO have been presented aiming to identify patient subgroups that may benefit from distal EVT.^19^ In a secondary analysis of DISTAL, Anastasiou et al. found that EVT plus BMT compared to BMT alone in patients with a bigger mismatch volume led to better mRS at 90 days and additionally to a bigger volume of saved brain tissue. Another analysis of DISTAL investigated 85 cases and found that successful first-pass reperfusion was 63.5% overall, with 55.6% for the triaxial ADAPT and 69.4% for the quadriaxial QUATTRO-ADAPT, concluding that ADAPT, especially in a quadriaxial approach, was effective and safe. In a subanalysis of ESCAPE, MeVO including 529 participants of the intention-to-treat population CTA-based collateral status was the only imaging variable associated with the primary outcome in uni-variable analysis.^19^

Future directions in this field must therefore focus on refinement. Improved patient stratification using advanced imaging modalities to better identify target occlusions, assess tissue viability accurately in distal territories, and evaluate collateral status will be critical.^6,16^ Furthermore, the development and rigorous testing of novel thrombectomy devices specifically designed for the smaller caliber and tortuosity of distal vessels are essential. Ultimately, future research must aim to identify specific subgroups within the heterogeneous DMVO population—potentially defined by occlusion location (e.g. very distal vs. more proximal M2), clot characteristics, imaging profile, or clinical severity—who might genuinely benefit from a refined, safer endovascular approach, moving beyond the broad inclusions that led to the neutral results of recent trials.

## Conclusion

In conclusion, the landscape of EVT for DMVOs has been significantly reshaped by the recent negative findings from the DISTAL, ESCAPE-MeVO, and DISCOUNT RCTs. Despite the compelling rationale extrapolated from the success of EVT in LVO, these rigorous studies consistently failed to demonstrate a benefit in functional outcomes when EVT was added to BMT for a broad population of patients with DMVO strokes. Moreover, the trials highlighted potential safety concerns, with trends towards increased risks of sICH and mortality associated with the endovascular procedure in this specific context.

The reasons for this lack of benefit are likely multifactorial, stemming from the inherent challenges of intervening in the distal cerebral vasculature. Increased procedural risks due to smaller vessel caliber, tortuosity, and fragility, potentially exacerbated by the use of devices primarily designed for LVOs, appear to contribute significantly. Concurrently, the heterogeneity of DMVO strokes, difficulties in precise imaging-based patient selection, and the considerable effectiveness of modern BMT, including intravenous thrombolysis, likely narrowed the potential margin of benefit for EVT in the populations studied.

Based on the current high-level evidence, routine EVT for unselected patients with DMVO cannot be recommended and should not be considered a default care pathway. Adherence to evidence-based guidelines is paramount, emphasizing the need for caution before adopting interventions lacking proven efficacy, particularly when associated with potential harm. However, the neutral trial results do not necessarily signify the end of the road for thrombectomy in all distal occlusions. Techniques like SPORNS, designed for very distal targets, show preliminary promise in a specific niche, suggesting that procedural refinement and adaptation are crucial. The path forward requires a more nuanced approach, focusing on meticulous research to identify specific patient subgroups—potentially defined by advanced imaging characteristics, precise occlusion location, or clinical profile—who might derive benefit from EVT performed with optimized techniques and dedicated distal devices. Continued innovation and rigorous investigation are essential to determine if and how the frontiers of thrombectomy can be safely and effectively extended into the more challenging territories of the distal cerebral vasculature.
